# Clinical and Molecular Data Define a Diagnosis of Arrhythmogenic Cardiomyopathy in a Carrier of a Brugada-Syndrome-Associated *PKP2* Mutation

**DOI:** 10.3390/genes11050571

**Published:** 2020-05-20

**Authors:** Simone Persampieri, Chiara Assunta Pilato, Elena Sommariva, Angela Serena Maione, Ilaria Stadiotti, Antonio Ranalletta, Margherita Torchio, Antonio Dello Russo, Cristina Basso, Giulio Pompilio, Claudio Tondo, Michela Casella

**Affiliations:** 1 Cardiac Arrhythmia Research Centre, Centro Cardiologico Monzino IRCCS, 20141 Milan, Italy; simone.persampieri@gmail.com (S.P.); remoantonio_ranalletta@hotmail.it (A.R.); antonio.dellorusso@gmail.com (A.D.R.); claudio.tondo@ccfm.it (C.T.); michela.casella@ccfm.it (M.C.); 2 Vascular Biology and Regenerative Medicine Unit, Centro Cardiologico Monzino IRCCS, 20141 Milan, Italy; chiarapilato91@gmail.com (C.A.P.); angela.maione@ccfm.it (A.S.M.); ilaria.stadiotti@ccfm.it (I.S.); giulio.pompilio@ccfm.it (G.P.); 3 Istituto Auxologico Italiano, IRCCS, Center for Cardiac Arrhythmias of Genetic Origin and Laboratory of Cardiovascular Genetics, 20149 Milan, Italy; m.torchio@auxologico.it; 4 Cardiology and Arrhythmology Clinic, University Hospital “Ospedali Riuniti Umberto I–Lancisi - Salesi”, 60020 Ancona, Italy; 5 Cardiovascular Pathology Unit, Department of Cardiac-Thoracic-Vascular Sciences and Public Health, University of Padua, 35128 Padua, Italy; cristina.basso@unipd.it; 6 Department of Clinical Sciences and Community Health, Università degli Studi di Milano, 20122 Milan, Italy

**Keywords:** Brugada syndrome, arrhythmogenic cardiomyopathy, *PKP2*, endomyocardial biopsy, cardiac mesenchymal stromal cells, mutation, diagnosis, functional studies

## Abstract

Plakophilin-2 (*PKP2*) is the most frequently mutated desmosomal gene in arrhythmogenic cardiomyopathy (ACM), a disease characterized by structural and electrical alterations predominantly affecting the right ventricular myocardium. Notably, ACM cases without overt structural alterations are frequently reported, mainly in the early phases of the disease. Recently, the *PKP2* p.S183N mutation was found in a patient affected by Brugada syndrome (BS), an inherited arrhythmic channelopathy most commonly caused by sodium channel gene mutations. We here describe a case of a patient carrier of the same BS-related *PKP2* p.S183N mutation but with a clear diagnosis of ACM. Specifically, we report how clinical and molecular investigations can be integrated for diagnostic purposes, distinguishing between ACM and BS, which are increasingly recognized as syndromes with clinical and genetic overlaps. This observation is fundamentally relevant in redefining the role of genetics in the approach to the arrhythmic patient, progressing beyond the concept of “one mutation, one disease”, and raising concerns about the most appropriate approach to patients affected by structural/electrical cardiomyopathy. The merging of genetics, electroanatomical mapping, and tissue and cell characterization summarized in our patient seems to be the most complete diagnostic algorithm, favoring a reliable diagnosis.

## 1. Introduction

Arrhythmogenic cardiomyopathy (ACM) and Brugada syndrome (BS) are inherited arrhythmia diseases that lead to sudden cardiac death. ACM hearts are characterized by ventricular structural alterations showing a progressive fibro-fatty infiltration associated with ventricular mechanical and electrical dysfunction. Mutations in different genes, mainly desmosomal, have been implicated in ACM pathogenesis, and most reside in the gene coding for Plakophilin-2 (*PKP2*) [1]. BS is a genetic channelopathy characterized by ST-segment elevation of coved morphology in right precordial electrocardiogram (ECG) leads, increased risk of ventricular tachycardia, and fibrillation in the absence of cardiac structural disease [2]. Mutations in the *SCN5A* gene, coding for the main cardiac sodium channel, account for approximately 20%–25% of genotype-positive subjects, and overall only 25%–30% of BS patients have a known genetic defect [3].

The intercalated disc hosts a common protein interacting network, the connexome, that includes molecules of the desmosome and the voltage-gated sodium channel (VGSC) complex. According to this, if the molecular substrates (desmosomes and VGSC) are part of a common network, BS and ACM should also share some common features. It is estimated that as many as 70% of the mutations linked to familial ACM are in the gene coding for *PKP2*, a 98 kDa desmosomal protein that interacts with plakoglobin, desmoplakin, and the desmosomal cadherins via its amino-terminal domain [4]. Mutations in *PKP2* may therefore destabilize the desmosome and result in arrhythmias and structural alteration simultaneously.

Although a general phenotypic distinction exists between the two pathologies, imaging and histopathological data support the notion that BS is not purely arrhythmogenic but includes, in some cases, structural anomalies [5]. Molecular data demonstrated that arrhythmias in ACM are consequent not only to tissue alterations but also to changes in the intercalated disc subdomain, including desmosomes, connexins, and sodium channels [6]. Additionally, from a genetic point of view, overlapping between ACM and BS was reported. In the continuing search for new causative gene variants in genetically-negative patients, researchers identified *SCN5A* mutations in some ACM patients [7], and *PKP2* mutations were associated with BS [8]. The two diseases seem to overlap in more than one aspect and a deeper analysis of every patient is required. Herein, we present a patient diagnosed with ACM, in whom a *PKP2* mutation, known to be causative for BS, was found. In particular, we report how molecular data (based on the use of cardiac mesenchymal stromal cells (C-MSCs) as an ACM in vitro model [9]) could confirm the clinical correct diagnosis.

## 2. Materials and Methods 

### 2.1. Ethical Statement

This study complied with the Declaration of Helsinki and was approved by the Centro Cardiologico Monzino-IRCCS Ethic Committee. Written consent was signed by participating patients.

### 2.2. Genotype Analysis

DNA was extracted from blood. Next-generation sequencing was performed (Illumina NextSeq, San Diego, CA, USA) with the TruSight™ Cardio Sequencing Kit. The alignment of sequence reads to reference human genome (GRCh37/hg19) was performed using GATK software (the GATK software is available as an open-source framework on The Broad Institute’s website). Variants in *DSC2*, *DSG2*, *DSP*, *PKP2*, *JUP*, *TMEM43*, *RYR2*, *PLN*, *SCN5A*, and *LMNA* were filtered with Wannovar and classified according to [10]. Pathogenic mutations were confirmed by Sanger sequencing.

### 2.3. Cardiac Mesenchymal Stromal Cell isolation

Cells were obtained from patient endomyocardial biopsies, as previously described [11]. 

### 2.4. Oil Red O Staining

C-MSCs were cultured in adipogenic medium (as in [11]) for three days, fixed with 4% paraformaldehyde (PFA) and neutral lipids were visualized by Oil Red O (ORO) staining. Pictures were captured with an Axiovert microscope (Zeiss, Oberkochen, Germany) and quantified with AxioVision Rel.4.8. (Zeiss, Oberkochen, Germany).

### 2.5. Real-Time PCR

Total RNA was extracted using Trizol (ThermoFisher Scientific, Waltham, MS, USA) and reverse transcribed with SuperscriptIII First-Strand Synthesis SuperMix (Invitrogen, Carlsbad, CA, USA). Quantitative real time-polymerase chain reaction (qRT-PCR) was performed in duplicate using 10 ng of cDNA, the iTaq Universal SYBR Green Supermix (Bio-Rad, Hercules, California, United States) and the following primers:

*PLIN1-FW*: 5′-CATTGAGAAGGTGGTGGAGTA-3′

*PLIN1-REV*: 5′-CTTGGCCTTGGGAGACTT-3′

*PPARγ-FW*: 5′-ACATAAAGTCCTTCCCGCTGACCA-3′

*PPARγ-REV*: 5′-AAACTGGCAGCCCTGAAAGATGC -3′

*GAPDH- FW*: 5′- ATGTTCGTCATGGGTGTGAA-3′

*GAPDH- REV*: 5′- GTCTTCTGGGTGGCAGTGAT-3′

### 2.6. Western Blot Analysis

C-MSC proteins were separated and transferred as in [9]. The membrane was incubated with primary antibodies against Plakophilin-2 (PKP2; BD Transduction Laboratories; Franklin Lakes, NJ, USA; 1:500), and Glyceraldehyde 3-phosphate dehydrogenase (GAPDH; Santa Cruz Biotechnology; Dallas, TX, USA; 1:2000). After incubation with proper secondary chemiluminescent antibodies, the signal was acquired and quantified using Alliance software (version 1D MAX, UVITEC, Cambridge, England, UK).

### 2.7. Immunofluorescence

After fixation with 4% paraformaldehyde (PFA), cells were incubated with primary antibodies against plakoglobin (PG; Sigma-Aldrich; St. Louis, Missouri, United States; 1:100) and peroxisome proliferator-activated receptor γ (PPAR-γ; Santa Cruz Biotechnology; Dallas, TX, USA; 1:100). Cells were incubated with the appropriate Alexa 488-conjugated secondary antibody. Cell nuclei were stained with Hoechst 33342. Pictures were acquired with an Apotome microscope (Zeiss, Oberkochen, Germany). The positive cell number was normalized to the nuclei number.

### 2.8. Statistical Analysis

Statistics were performed using GraphPad Prism software (version 5 San Diego, CA, USA). Comparison between groups was performed using two-tailed Student’s *t*-tests. Results were considered statistically significant for *p* < 0.05.

## 3. Results

### 3.1. Clinical Data

**Figure 1:**
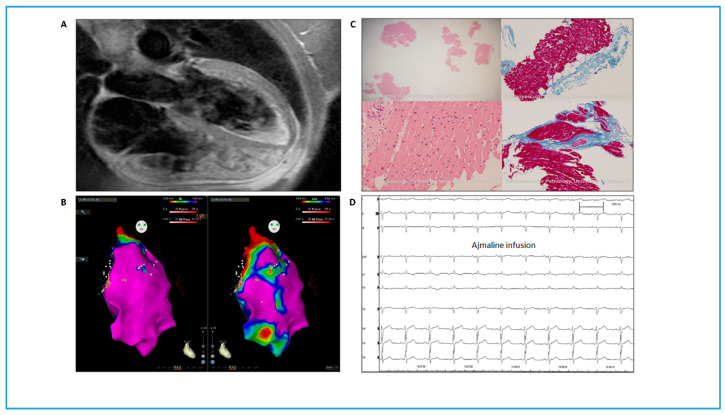
Clinical data are not compatible with BS phenotypes. (**A**) Cardiac magnetic resonance showing biventricular dilation and areas suspicious for adipose infiltration at the apex of the right ventricle (RV). (**B**) Bipolar (left) and unipolar (right) electroanatomical mapping of the RV of the patient. Unipolar voltage mapping showed low-voltage areas in the RV outflow tract. (**C**) Endomyocardial biopsy showing fibro-fatty substitution, compatible with arrhythmogenic cardiomyopathy diagnosis. (**D**) 1 mg/kg of ajmaline was administered to the patient over 10 min followed by 5 min of wash-out observation: no type-1 BS electrocardiographic pattern was induced. BS, Brugada syndrome.

A 41-year-old man, known for a history of premature ventricular complexes (PVCs) since 2009, with no prior history of cardiac diseases and no family history of sudden death, was admitted to our department in 2016. A basal ECG showed sinus bradycardia, nonspecific repolarization abnormalities. Previous echocardiogram and cardiac magnetic resonance (MRI) showed cardiac biventricular dysfunction with enlargement of the right-side chambers. No areas of late gadolinium enhancement or lipomatous infiltration were evident. A two-dimensional echocardiogram at admission showed biventricular dilation (left ventricular end-diastolic volume (LVEDV), 80 mL/m^2^; right ventricular end-diastolic basal diameter, 45 mm) and mild biventricular dysfunction (left ventricular ejection fraction (LVEF), 50%; tricuspid annular plane systolic excursion (TAPSE), 19 mm; right ventricular fractional area change (RVFAC), 21%), with no relevant valvular abnormalities. A cardiac MRI was performed again during hospitalization (Figure 1A) and showed biventricular dilation (LVEDV, 125.8 mL/m^2^; right ventricular end-diastolic volume (RVEDV), 171 mL/m^2^), mild biventricular dysfunction (LVEF, 50%; right ventricular ejection fraction (RVEF), 37%), right ventricle diffuse hypokinesia with basal right ventricle outflow tract (RVOT) akinesia, and areas suspicious for adipose infiltration at the apex of the right ventricle and in the basal segments of the anterior wall of the left ventricle. A Holter ECG showed 3500 premature ventricular contractions (PVCs), with 223 couplets and 13 triplets. No sustained ventricular arrhythmias were evoked by programmed stimulation (electrophysiological study (EPS)). During the EPS, three different PVC morphologies were recorded: one with RVOT morphology and two with left-side origins. According to the substrate analysis and the RVOT morphology of the PVC, pace mapping was performed, and finally radiofrequency was applied to ablate the origin of the PVC. Electroanatomical mapping (Figure 1B) showed unipolar low-voltage areas in the RVOT, which were suggestive of epicardial scarring [12]. In the same area, an endocardial cardiac biopsy was performed, which was sent for histological analysis. The results confirmed the tissue pattern was compatible with the diagnosis of ACM (fibro-fatty substitution, Figure 1C). The ACM diagnosis was based on one major MRI criterion, one minor arrhythmia criterion, and one minor wall-tissue characterization criterion [13]. An implantable cardioverter-defibrillator (ICD) was therefore implanted. The patient was then discharged with sotalol therapy. The genetic screening by next-generation sequencing of the genes *DSC2*, *DSG2*, *DSP*, *PKP2*, *JUP*, *TMEM43*, *RYR2*, *PLN*, and *LMNA* revealed the presence of the *PKP2* gene in exon 3 of the c.548G>A mutation that causes p.S183N substitution. This rare variant was previously described as causative of BS [8], but has never been associated with ACM. This result raised the suspicion of a misdiagnosis and the patient was recalled to perform an ajmaline test. No type 1 Brugada ECG pattern was evoked (Figure 1D) and the diagnosis of BS was ruled out. During three years of follow-up, no recurrent arrhythmias were evident from remote monitoring or ambulatory visits. 

A 28-year-old athlete with a clinical and genetic diagnosis of BS (heterozygous c.4867C>T, p.Arg1623X in the *SCN5A* gene), who previously underwent cardiac biopsy to rule out ACM, was used for comparative analysis.

### 3.2. Molecular Data

**Figure 2:**
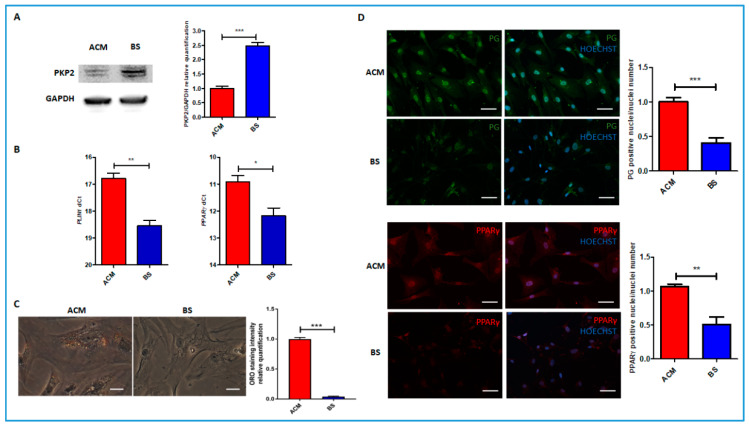
C-MSC data are distinctive of ACM. (**A**) Western blot shows higher *PKP2* expression in ACM than in BS C-MSCs cultured in basal condition. *GAPDH* is shown as a loading control and used to normalize the quantification. (**B**) qRT-PCR data showing higher *PLIN1* and *PPARγ* expression in ACM than in BS C-MSCs in adipogenic medium. (**C**) Higher ORO staining is detected in ACM vs. BS C-MSCs cultured in adipogenic medium for three days, as in the representative image and quantification. Scale bar is 20 μm. (**D**) Representative immunofluorescence images of C-MSCs cultured in adipogenic medium for three days and stained with PG or PPARγ antibodies. More positive nuclei/nuclei numbers were observed in ACM than in BS cells for both markers. Scale bar is 50 μm. The graphs show the mean and standard error of three technical replicates. *p < 0.05; **p < 0.01 ***p < 0.001 (two-tailed Student’s *t*-test). ACM, arrhythmogenic cardiomyopathy; C-MSC, cardiac mesenchymal stromal cells; GAPDH, glyceraldehyde 3-phosphate dehydrogenase; ORO, Oil Red O; PG, plakoglobin; *PKP2*, plakophilin 2; *PLIN1*, perilipin-1; *PPARγ*, peroxisome proliferator-activated receptor gamma.

C-MSCs were isolated from endomyocardial biopsies obtained from the ACM and BS patients and used to study their molecular phenotypes. As reported in the literature, PKP2 is expressed less in C-MSC isolated from ACM patients than in healthy controls [9]. Accordingly, the level of PKP2 in the ACM patient’s C-MSCs was lower than that of the C-MSCs obtained from the BS patient (Figure 2A).

We then investigated the capability of ACM and BS C-MSCs to accumulate lipids in adipogenic conditions. We found that ACM C-MSCs showed higher expressions of adipogenic genes such as *PLIN1* and *PPARγ* and were more prone to accumulate neutral lipids than C-MSCs in the BS patient (Figure 2B, C). It has been reported that switching to adipogenic fate in ACM cases is linked to alterations in the Wnt/β-catenin pathway and PG nuclear translocation [9]. ACM C-MSCs showed a larger number of PG-positive nuclei than BS C-MSCs as well as more *PPARγ* nuclear localization, as previously observed [14] when cultured in adipogenic medium (Figure 2D).

## 4. Discussion

Defining the meaning and the correct genotype–phenotype association of potentially pathogenic variants is an extreme challenge, even in the presence of in vitro functional validation. The effect of a mutation could be modified by various factors, such as the expression of the allele in different cell types, the patient’s genetic background (which may include phenotype-modifier variants), and the compensation of other mechanisms, including epigenetic and environmental mechanisms. 

To date, eight variants in the PKP2 gene—the most prevalent gene associated with ACM—have been found in BS patients, either alone or in combination with other gene variants [15,16,17]. In vitro studies in HL-1 cells of four of these variants showed sodium current alterations and a PKP2-dependent defect in sodium channel trafficking via microtubules to the intercalated disc [17]. We hypothesized that these mechanisms are shared between BS and ACM based on both also occurring in induced pluripotent stem cell (iPSC)-derived cardiomyocytes from a patient carrier of an ACM-associated *PKP2* mutation and in *PKP2*-deficient mouse cardiomyocytes.

However, the present report describes one of the *PKP2* BS-related mutations (p.S183N) associated with an ACM phenotype.

A limitation of the previous study [8] was the impossibility of determining whether the reported *PKP2* mutation occurred in the context of additional genetic differences, other than in the four most common genes associated with BS. Indeed, there is a large number of known proteins that can directly or indirectly affect the sodium current. The clinical characteristics of the patient carrier of the p.S183N mutation were not definitely diagnostic of BS, since the type 1 ECG pattern was observed during a febrile episode and not confirmed by a provocative test [8]. This finding could therefore be an unspecific sign of BS-like ECG [18]. Further clinical investigation should be performed to exclude the presence of ACM in the concealed phase mimicking BS. The ClinGen consortium has reclassified most of the genes described as potentially linked to BS as not having sufficient evidence to support their causality for BS [19]. Thus, genetic testing of these genes may lead to incorrect interpretation of variants.

Despite being recognized as functionally relevant [8], rare (minor allele frequency (MAF) = 0.0082 according to the Genome Aggregation Database) and relatively conserved among species, the available evidence cannot rule out a low pathogenicity effect of the *PKP2* p.S183N variant alone. It may represent a phenotypic modulator with no clear deterministic cut-off through either BS or ACM phenotypes. Few rare causes of ACM have not been excluded by our genetic tests and known genetic causes of ACM only support about 50% of affected patients.

In conclusion, *PKP2* variants in BS cases should be interpreted carefully. The complex decision-making network may benefit from additional functional analyses in cells that carry the whole genetic background of a patient, as in this present report, accompanied by a careful clinical and genetic interpretation in a family context.
